# Efficacy of Letrozole vs Clomiphene Citrate for induction of ovulation in women with polycystic ovarian syndrome

**DOI:** 10.12669/pjms.40.1.7971

**Published:** 2024

**Authors:** Tayyiba Wasim, Tahira Nasrin, Javeria Zunair, Sonia Irshad

**Affiliations:** 1 Tayyiba Wasim, FCPS, Department of Obstetrics and Gynaecology, Services Hospital Lahore, Lahore, Pakistan; 2 Tahira Nasrin, FCPS, Department of Obstetrics and Gynaecology, Services Hospital Lahore, Lahore, Pakistan; 3 Javeria Zunair, FCPS-I Department of Gynecology, Sheikh Zayed Hospital, Lahore, Pakistan; 4 Sonia Irshad, FCPS-I, Department of Obstetrics and Gynaecology, Services Hospital Lahore, Lahore, Pakistan

**Keywords:** Polycystic ovarian syndrome, Letrozole, Clomiphene citrate

## Abstract

**Objective:**

To compare the efficacy of letrozole vs Clomiphene citrate for ovulation induction in PCOS women.

**Methods:**

This double blind randomized controlled trial was conducted at Services Hospital, Lahore, from January 2016 to December 2020. Total 220 patients, diagnosed with PCOS according to Rotterdam criteria were randomly assigned into two groups after taking informed consent. The women were followed for ovulation, pregnancy and live birth rates in the next five consecutive menstrual cycles with either clomiphene citrate or letrozole.

**Results:**

Letrozole had significantly better pregnancy rate (29.0% vs 15.4% p-value 0.015), monofollicular development (77.2% vs 52.7% p-value 0.000) and live birth rate (25.4% vs 10.9% p-value 0.005) as compared to clomiphene citrate. There was no difference between the two groups in ovulation rate (68.1% vs 63.6%, p-value 0.477), early pregnancy loss (3.6% vs 4.5% p-value 0.734), and twin pregnancy (0.0% vs 1.81% p-value 0.155). There was no ectopic pregnancy and no congenital anomalies in both groups. Hot flushes were higher in clomiphene group (31.8% vs 12.7% p-value 0.001) while fatigue (30.9% vs 8.1% p-value 0.000) and dizziness (21.8% vs 10.0% p-value 0.029) was higher with letrozole but these were well tolerated.

**Conclusion:**

Letrozole is better treatment choice than clomiphene citrate in PCOS women with infertility in terms of pregnancy and live birth rate.

***ClinicalTrials.gov Identifier:*** NCT05702957.

## INTRODUCTION

Polycystic ovarian syndrome (PCOS) is the most common endocrine disorder which affects 6-20% of women of reproductive age group.^1^ It is characterized by reproductive and metabolic abnormalities and is the commonest cause of anovulatory infertility worldwide.^2^ In addition to life style changes, various drugs have been used to induce ovulation in these patients with infertility. Clomiphene citrate is a selective estrogen-receptor modulator which leads to ovarian stimulation. It has been used in PCOS women for infertility as first line ovulation induction drug.^3^ It is orally administered, easily available and inexpensive but associated with few drawbacks. The ovulation rates with clomiphene citrate are in the range of 60-85% but a conception rate of only 18-20%^4^. This is due to antiestrogenic action of clomiphene citrate on endometrium and cervical mucus. Other side effects like development of resistance to clomiphene, development of multiple follicles, ovarian hyper stimulation and formation of cysts are also worrisome.^5^

Letrozole is an aromatase inhibitor which has been used as second choice medical treatment in PCOS women especially who develop resistance with clomiphene.^6^ It inhibits the androgens conversion to estrogen which causes increase release of FSH from anterior pituitary due to decreased inhibitory influence of estrogen on hypothalamic-pituitary axis. Its action also leads to accumulated androgens in the ovary which further increase sensitivity of follicles to FSH resulting in monofollicular development. Unlike clomiphene citrate, letrozole does not have any anti estrogenic peripheral action therefore it does not adversely affect endometrial development or cervical mucus production. It also improves endometrial receptivity compared with clomiphene citrate and increased pregnancy rate has been reported with its use.^7^ Guidelines and recommendations for infertility treatment in PCOS women with infertility is lacking due to low quality of evidence.^8^ Generating evidence of best treatment of infertility associated with PCOS is important as infertility is a curse in low middle income countries (LMIC).

In Pakistan, clomiphene citrate is used as first line drug and local data about comparison of efficacy of letrozole and clomiphene citrate for treatment of PCOS in subfertility as first choice therapy in conflicting in nature.^9^,^10^ There is no randomized control trial reported from Pakistan which compares Letrozole with clomiphene with follow up of patients till delivery. The objective of our study was to compare efficacy of these two drugs in terms of live birth rate with the hope that the results would help to select more appropriate treatment for patients with infertility due to PCOS.

## METHODS

It was a double blind randomized controlled trial conducted in department of Gynecology & Obstetrics, Services Hospital Lahore from January 2016-December 2020.. To detect a clinical significant difference of 20% between previously reported pregnancy rate of clomiphene citrate and letrozole with a two sided 5% significance level, a sample of 220 participants (110 each arm) was required^4^,^5^. Sample size calculated was 220 patients but keeping in mind the drop outs of long treatment, 260 patients were assessed for eligibility.

### Inclusion & Exclusion Criteria

Patients with PCOS aged between 18-40 years and with normal husband semen analysis and normal tubal patency on hysterosalpigography or laparoscopy who failed to get pregnant with regular sexual intercourse with no contraception after 12 months or more were enrolled in the study. PCOS diagnosis depends upon Rotterdam criteria^8^ which includes presence of any two of the following characteristics: clinical or biochemical evidence of excess androgen, polycystic ovaries on ultrasound (12 or more follicles measuring 2-9mm in diameter or increased ovarian volume more than 10cm^3^) or oligomenorrhea. Patients having hyperprolactinemia, thyroid disease, male subfertility, endometriosis, and unexplained infertility were in exclusion criteria.

### Ethical Approval

The Services Hospital Lahore IRB gave ethical approval for the study on 23^rd^ January 2016 ref no. IRB/2015/209/SIMS

Informed consent was taken by all elgible patients after explaining about the trial and those who gave consent were enrolled. Two treatment groups were made by random computer-generated number i.e., Group A and Group B. Two Concealed boxes labeling A and B containing study drugs were made by pharmacist. Then participants were given drug from concealed boxes according to computer generated number. Group-A patients were given clomiphene citrate 50mg once daily as starting low dose from 2^nd^ day of menses for five days. Ovulation was monitored by trans-vaginal ultrasound follicular tracking from day 10-12 of menstrual cycle till an ovarian follicle achieved a diameter of 18-25mm and mid luteal (Day 21) progesterone measurement. If patient did not ovulate with 50 mg dose, then dose was increased by 50 mg increments uptil 150 mg (three tablets) a day. If patient achieved ovulation with whatever dose of clomiphene, the treatment was repeated for 5 more cycles with that dose. Group-B patients were given letrozole 2.5 mg tablet from 2^nd^ day of menses for 5 days. If patient failed to ovulate, the dose was increased to 5mg (two tablets) to a maximum of 7.5mg (three tablets). The treatment was repeated for five more cycles with the dose that achieved ovulation and monitored for pregnancy.

In addition, both treatment groups were advised lifestyle modification which includes well balanced diet and regular exercise. Trans-vaginal ultrasound monitoring done from day 10-12 of menstrual cycle till an ovarian follicle achieved a diameter of 18-25mm. Women who failed to ovulate despite maximum dose of each drug were declared non responders and were counselled for other options of treatment like gonadotrophin injections or in vitro fertilization.

When at least one ovarian follicle achieved a diameter of 18-25 mm, then injection HCG 10,000 IU IM was given. Patients were counseled for timed intercourse after 24-36 hours of HCG injection. Ovulation was confirmed by day 21 serum progesterone. After one week of missed periods, urine pregnancy test was done to confirm the pregnancy. TVS was done to confirm ongoing pregnancy. All patients who conceived, were followed up till delivery. Live birth rate was primary outcome measure while secondary outcomes were pregnancy, mono-follicular vs multi-follicular rate, ovulation rate, multiple pregnancies, miscarriage, ectopic pregnancy, congenital anomalies and adverse events (hot flushes, fatigue and dizziness).

### Statistical Analysis

All data were entered in SPSS version 23 and analyzed. For comparison Chi- square test was used between qualitative variables. <0.05 P-value was considered statistically significant.

## RESULTS

**Fig.1:**
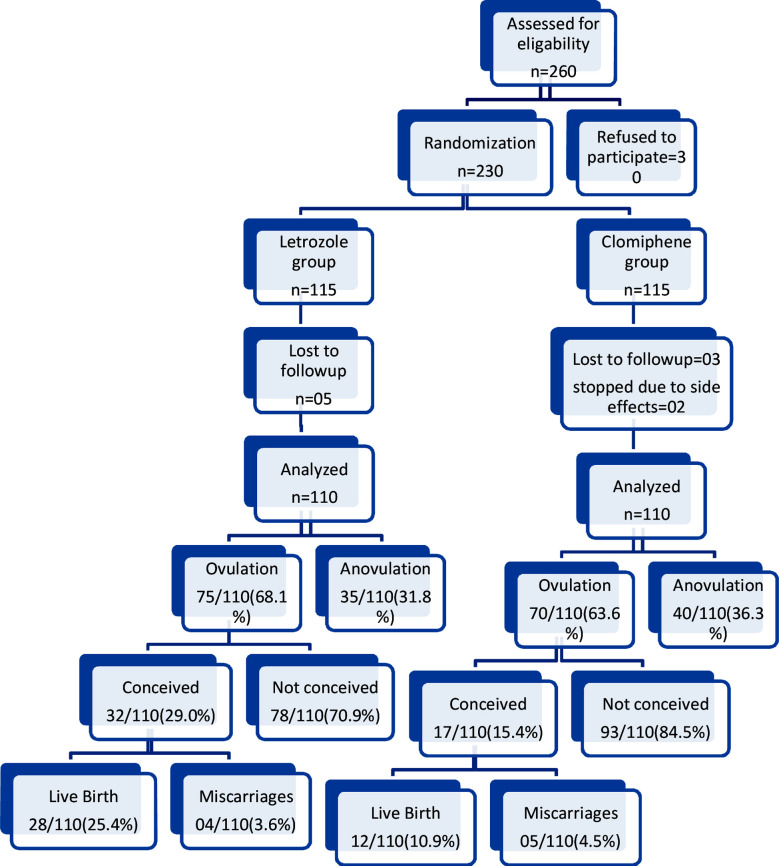
CONSORT Flow Chart of the trial.

Flow of participants through the trial shows in Fig.1. A total of 260 eligible women were invited to participate of which 230 were randomized. Out of five patients in letrozole group and three patients in clomiphene group were lost to follow up after first cycle, two patients refused to continue clomiphene because of nausea and 220 patients completed the follow-up and were analyzed. The baseline characteristics of patients in both groups were well matched regarding age, BMI, infertility period, occupation, menstrual cycle pattern, clinical and ultrasound features of PCOS, (Table-I).

**Table-I:** Baseline Characteristics of Patients.

Characteristics	Letrozole Group (N=110)	Clomiphene citrate Group(N=110)	P-value
Age	24.86±4.21	25.43±4.39	0.212
BMI	27.1±4.92	28.4±4.95	0.451
***Occupation***			0.668
Working	35(31.8%)	38(34.5%)	
Housewife	75(68.1%)	72(65.4%)	
***Subfertility***			0.496
Primary subfertility	65(59.09%)	60(54.54%)	
Secondary infertility	45(40.9%)	50(45.45%)	
Infertility duration (years)	4.5±1.5	4.4±1.6	0.729
***Menses***			0.349
Oligomenorrhoea	65(59.09%)	59(53.63%)	
Amenorrhoea	15(13.63%)	10(9.09%)	
Irregular	25(22.72%)	32(29.09%)	
Regular	5(4.54%)	9(8.18%)	
Hirsutism	45(40.9%)	37(33.63%)	0.265
Acne	31(28.18%)	37(33.63%)	0.381
USG			0.561
Polycystic ovaries	93(84.54%)	96(87.27%)	

Regarding outcome, Letrozole was associated with significantly higher pregnancy (29.0% vs CC 15.4% p=0.015) and live birth rate (25.4% vs 10.9% p=0.005). Although there was no difference between the two groups in ovulation rate (68.1% vs 63.6% p-value 0.477)). In clomiphene group out of 70(63.6%) patients who ovulated, 40 patients (57.14%) ovulated with a 100mg dose, 19(27.14%) with150mg dose and 11(15.71%) patients with a 50mg dosage while in letrozole group out of 75(68.1%) patients who ovulated, 38 patients (50.67%) ovulated with a 5mg dose, 20(26.67%) with 7.5mg dose and 17(22.67%) patients with a 2.5mg dosage. Out of 75(68.1%) who ovulated with letrozole, 32(29.0%) successfully became pregnant. Among them, 9(28.1%) patients conceived in second cycle, 15(46.88%) in third cycle, 7(21.88%) in fourth and 1(3.13%) in fifth cycle of treatment. Out of 70 patients who ovulated with clomiphene, 17 (15.4%) patients successfully became pregnant. Among them,1(5.88%) conceived in first cycle, 4(23.53%) in second, 4(23.53%) in third and 8(47.06%) in fourth cycle of treatment. Letrozole group was associated with statistically significantly higher monofollicular development (77.2% vs 52.7% p-value 0.00). There was no difference in early pregnancy loss in both groups (letrozole 3.6% vs 4.5% p= 0.734).

There were no congenital anomalies and ectopic pregnancy. There was statistically no significant difference between the two groups in twin pregnancy (0.0 vs 1.81% p= 0.155). Regarding side effects, hot flushes were significantly higher in clomiphene citrate group (CC 31.8% vs letrozole 12.7% p-value 0.001) while incidence of fatigue (30.9% vs 8.1% p-value 0.000) and dizziness (21.8% vs 10.0% p-value 0.029) was significantly higher with letrozole, (Table-II).

**Table-II:** Outcomes with regard to live birth, ovarian stimulation, ovulation, pregnancy, pregnancy loss.

Outcomes	Letrozole Group (N=110)	Clomiphene Group (N=110)	P-value
***Ovarian stimulation***			
Monofollicular development	85(77.2%)	58(52.7%)	0.000
Multifollicular development	25(22.7%)	52(47.2%)	0.000
Ovulation rate	75(68.1%)	70(63.6%)	0.477
Pregnancy rate	32(29.0%)	17(15.4%)	0.015
Live birth rate	28(25.4%)	12(10.9%)	0.005
***Complications***			
Early pregnancy loss	4(3.6%)	5(4.5%)	0.734
Multiple pregnancy	0	2(1.81%)	0.155
Congenital anomalies	0	0	
Ectopic pregnancy	0	0	
***Side effects***			
Hot flushes	14(12.7%)	35(31.8%)	0.001
Fatigue	34(30.9%)	9(8.1%)	0.000
Dizziness	24(21.8%)	11(10.0%)	0.029

## DISCUSSION

It was first randomized control trial with live birth outcome comparing two treatments reported from Pakistan with fewer drop outs. In this study, clomiphene citrate group has mean BMI of 28.4±4.95 kg/m2 and letrozole group has mean BMI of 27.1±4.92 kg/m2. It has been established that 35-65% of PCOS patients are obese.^11^ Our patients had BMI of 27.1 kg/m2 and 28.4 kg/m2 in both groups which is comparable to studies Sidra S et al and Khakwani M et al from Pakistan.^9^,^10^ However, a study by Legro et al from United States has reported BMI of 35 in his patients.^12^ Elevated BMI is a major impediment for successful fertility treatment in these women. They respond poorly to ovulation induction and require higher doses of drugs. Abdominal adiposity increases insulin resistance. High insulin levels increase androgen production hence hair growth and acne. Even modest weight loss of 5% often results in improvement in reproductive and metabolic features of PCOS.^13^

In this study, letrozole was found effective and better choice of treatment for subfertility than clomiphene in PCOS women. Regarding primary outcome, letrozole treatment resulted in the higher live birth rate compared to CC (25.4%vs 10.9% p= 0.005). Similarly, pregnancy rate was higher with letrozole (29.0% vs 15.4% p= 0.015) despite similar ovulation rate. Clomiphene citrate has traditionally been used as the first line ovulation induction drug for patients with PCOS. The other drugs like letrozole and gonadotropins were tried as second line drugs in patients who did not respond to clomiphene. First double blind multicentric trial of 750 patients was reported by Legro et al showing better ovulation rate and cumulative birth rates in patients who received letrozole^12^. Following this, other RCTs were reported supporting letrozole being better in achieving pregnancy and live birth.^5^,^6^,^14^-^16^ Liu C et al. and Saha J et al. recommend no significant difference in ovulation and pregnancy rate in RCT with both drugs and recommend clomiphene citrate as first line drugs^15^,^18^. Wang R et al. and Roque M et al. in meta-analysis comprising of 1284 and 3962 patients have demonstrated that Letrozole use resulted in significantly better clinical pregnancy and live birth rate compared to clomiphene citrate in women with PCOS.^19^,^20^ This is due to the fact that clomiphene citrate has a long half-life of two weeks and its anti-estrogenic effects causes unfavorable effect on the quality and the amount of cervical mucus in addition to thinning of endometrium leading to implantation failure.

In our study, the ovulation rate was not statistically significantly different between the two study groups (CC 63.6%, Letrozole 68.1%, p= 0.47) which is similar to the studies by Zeba D et al and Liu C et al.^14^,^15^ While Amer SA et al, Khakwani M et al., Najafi PZ et al. and Sakar et al. reported significantly better ovulation in letrozole group.^6^,^10^,^16^,^17^ One of the issues with ovulation induction drugs is chances of multiple follicular development and hyperstimulation. Treatment with Clomiphene has been reported to be associated with 10-12% chances of having twins compared to 3-5% with letrozole.^5^,^14^ Aromatase inhibitors are selective estrogen receptor modulator, do more physiological hormonal stimulation of endometrium, lower multiple pregnancy rate due to single follicular recruitment. Letrozole showed highly statistically significant monofollicular development (77.2% vs 52.7% p-value 0.000) in our study. Bansai S et al. also reported significant more monofollicular development in patients taking letrozole as compared to clomiphene (68.4%44.8% p=0.00).^21^

PCOS patients has higher miscarriage rate than normal population.^2^-^4^ The reason is abnormality in endometrial reception, corpus luteum insufficiency, poor quality of ovum, insulin resistance development and increased androgen levels in body. In our study, there was no difference in miscarriage rate in both groups (letrozole 3.6% vs CC 4.5% p-value 0.734). Franik S et al in Cochrane data base of systematic reviews reported similar results.^5^ There were no congenital anomalies in our patients. Letrozole group safety data was an issue regarding fetal anomalies due to which clinicians were hesitant to use it but the meta-analysis of RCTs by Wang R et al and Roque M et al. have found no increase in neonatal/fetal anomalies in patients using letrozole as compared to spontaneous conception.^19^,^20^ Studies by Sharma S et al of 500 babies and Akbari Sene A et al. comparing 1237 CC cycles and 772 letrozole cycles have found congenital anomalies similar to general population and no significant difference was seen in both groups.^22^,^23^

Regarding side effects in our study, there was significantly higher hot flushes (31.8%) with clomiphene citrate while letrozole had significantly higher incidences of dizziness (21.8%) and fatigue (30.9%), but these were well tolerated by the patients and no serious adverse effects were observed. The side effects with clomiphene citrate of blurred vision, headache, nausea and vomiting are reported by Nahid L et al without any complication.^24^

The results of this trial are consistent with the Cochrane systematic review. However, with its robust design and follow up of patients till delivery provides compelling local evidence for superiority of letrozole over clomiphene as primary ovulation inducing agent in PCOS women with significantly better pregnancy and live birth rate. This has important implications in our population where infertility is largely treated by general practitioners and quacks and they use clomiphene as first line drug. This trial will help them change their practice and ensure better care of patients.

### Limitations

It was a single center study. We didn’t do life style modifications before treatment although they have proven to have better results. Further studies with a greater number of patients at multiple centers is recommended for patients of PCOS with infertility.

## CONCLUSION

Letrozole is better treatment choice than clomiphene citrate in PCOS women with infertility in terms of higher pregnancy rate and live birth rate. Letrozole should be considered as first-line treatment in anovulatory PCOS infertile patients.

### Authors’ Contribution:

**TW:** Conceived, designed, final manuscript editing and is responsible for integrity of research.

**TN:** Did initial manuscript writing.

**JZ** & **SI:** Did data collection and statistical analysis.
